# Genotyping an immunodeficiency causing c.1624–11G>A *ZAP70* mutation in Canadian Mennonites

**DOI:** 10.1186/s12881-016-0312-4

**Published:** 2016-07-22

**Authors:** M. L. Schroeder, B. Triggs-Raine, T. Zelinski

**Affiliations:** Department of Pediatrics and Child Health, Max Rady College of Medicine, Rady Faculty of Health Sciences, University of Manitoba, Winnipeg, Manitoba Canada; Department of Biochemistry and Medical Genetics, Max Rady College of Medicine, Rady Faculty of Health Sciences, University of Manitoba, Winnipeg, Manitoba Canada

## Abstract

**Background:**

Primary immunodeficiency is a life-threatening genetic disease that appeared to have an increased incidence in Manitoba Mennonites. Determining the genetic basis of this immunodeficiency was an essential step for providing early and appropriate medical intervention.

**Methods:**

Initially, DNA from probands affected with primary immunodeficiency and their family members was assessed for linkage to genes previously associated with immunodeficiency. Candidate genes were sequenced to identify the causative mutation. The frequency of the mutation among first and second degree relatives, as well as apparently unrelated community members was analyzed using a PCR-based assay.

**Results:**

A previously described c.1624–11G>A mutation in *ZAP70* was identified as the causative mutation in all affected probands that were analyzed. Among 125 study participants of Mennonite descent, 79 genotyped as normal, 39 were carriers and seven were affected. None of 115 non-Mennonite random individuals carried the mutation, whereas one of ten random DNA samples from individuals who self-identified as Mennonite was a carrier.

**Conclusions:**

In collaboration with the target community, we have developed a robust screening test for determining *ZAP70* genotype. Early identification of affected individuals has provided an opportunity for timely clinical intervention, while carrier identification has allowed for genetic counselling of at risk couples.

## Background

Primary immune deficiencies comprise a broad spectrum of diseases, ranging from severe combined immunodeficiency syndrome (SCID) which is uniformly lethal without a stem cell transplant, to several milder forms [1]. Among these is an autosomal recessively inherited ZAP-70 (zeta-chain associated protein kinase 70) deficiency, which despite having a less severe clinical phenotype, typically results in death by two years of age due to overwhelming sepsis. Arpaia et al., [2] defined the underlying mutation in three patients of Mennonite descent as a homozygous intronic *ZAP70* single nucleotide substitution (c.1624–11G>A). This mutation creates a new acceptor splice site, resulting in the insertion of nine nucleotides to the mRNA. The altered protein product is unstable, leading to a complete loss of kinase activity. Since ZAP-70 kinase is involved in T cell receptor signaling and is critical for T cell maturation, loss of function mutations lead to an absence of CD8^+^ T cells and inactive CD4^+^ T cells [2–5].

Mennonites are Anabaptists who descended from Swiss, Dutch and German ancestors. Persecuted for their religious beliefs and pacifism, Mennonite communities settled in various parts of Europe, seeking regions that were either sympathetic to, or tolerant of, their way of life. The first Mennonites (Swiss/German) immigrated to North America in the late 18^th^ century, settling primarily in Southern Ontario and Pennsylvania [6]. The Dutch/German Mennonites eventually settled in Russia and then immigrated to the Canadian prairies in two major waves, with about 7,000 immigrants arriving in the early 1870s, and another approximately 20,000 after World War I (1922 - 1930) [7].

For cultural and religious reasons Mennonites constitute a genetic isolate, where founding alleles continue to be expressed in the current population. We identified the *ZAP70* c.1624–11G>A mutation as the cause of a primary immunodeficiency in an extended Mennonite kindred. Prompted by the local Mennonite community and because timely diagnosis and treatment [8] are essential to reducing mortality due to ZAP-70 kinase deficiency, we developed a screening test which identified the c.1624–11G>A mutation as a frequent cause of immunodeficiency in this population.

## Methods

### Subjects

In total, 125 Mennonite subjects were enrolled in our study. Initially all test subjects were first or second degree relatives (105 individuals) of an affected individual. As the results became available to the family members, 20 other individuals of the broader community volunteered samples for *ZAP70* genotyping. These included young married couples expecting their first child, newly married couples (including two spouses of successfully treated affected individuals) and unmarried young adults. DNA from 125 random individuals, 10 of whom self-reported as Mennonite, was also used in this study.

### Clinical Synopsis

All affected children were well at birth, and presented between the ages of 4 and 17 months. Two were diagnosed shortly after birth before they became unwell because of an affected sibling. The method of presentation varied, however recurrent respiratory infection was seen in all of them. Pneumocystis jirovecci pneumonia requiring ventilation was the presenting symptom in one at the age of 4 months. A generalized failure to thrive, skin rash, enteritis, feeding difficulties, otitis media, thrush and pneumonia were seen with variable severity in all. B- and T-lymphocytes were present within the normal range in all patients, however the CD8+ count was decreased in all. The absence of a T-cell response to phytohemagglutinin was universal. Immune function was reconstituted by hematopoietic stem cell transplantation and all seven patients are currently alive and well (3 to 24 years post-transplant).

### Sanger Sequencing

Sanger sequencing was performed at The Centre for Applied Genomics, (The Hospital for Sick Children, Toronto, Canada), using the same primers and under the same conditions as were used for genotyping. The DNA sequence was determined for both strands of all fragments. The sequences obtained were compared with the reference allele sequence *ZAP70* (GenBank Accession number: NG_007727.1 for gDNA).

### c.1624–11G>A *ZAP70* Genotyping

Genomic DNA isolated from whole or cord blood (50–100 ng) or buccal swab (20–50 ng), was PCR amplified for three min at 95 °C for one cycle, one min at 95 °C, one min at 59 °C and 2 min at 72 °C for 30 cycles, followed by 10 min at 72 °C for one cycle, using the forward (5′-GTGATGCCCGACTGGATG-3′) and the reverse (5′-GGCTTTGGGTGAGATGACA-3′) primers. An aliquot of the resulting 534 bp product was incubated at 37 °C for 3 hr with 10U AluI. Products were resolved on a 2 % agarose gel for 1 hr at 150 V. Each genotyping result was determined by comparison, on the same gel, with DNA from known normal, carrier and affected individuals.

## Results

**Fig. 1:**
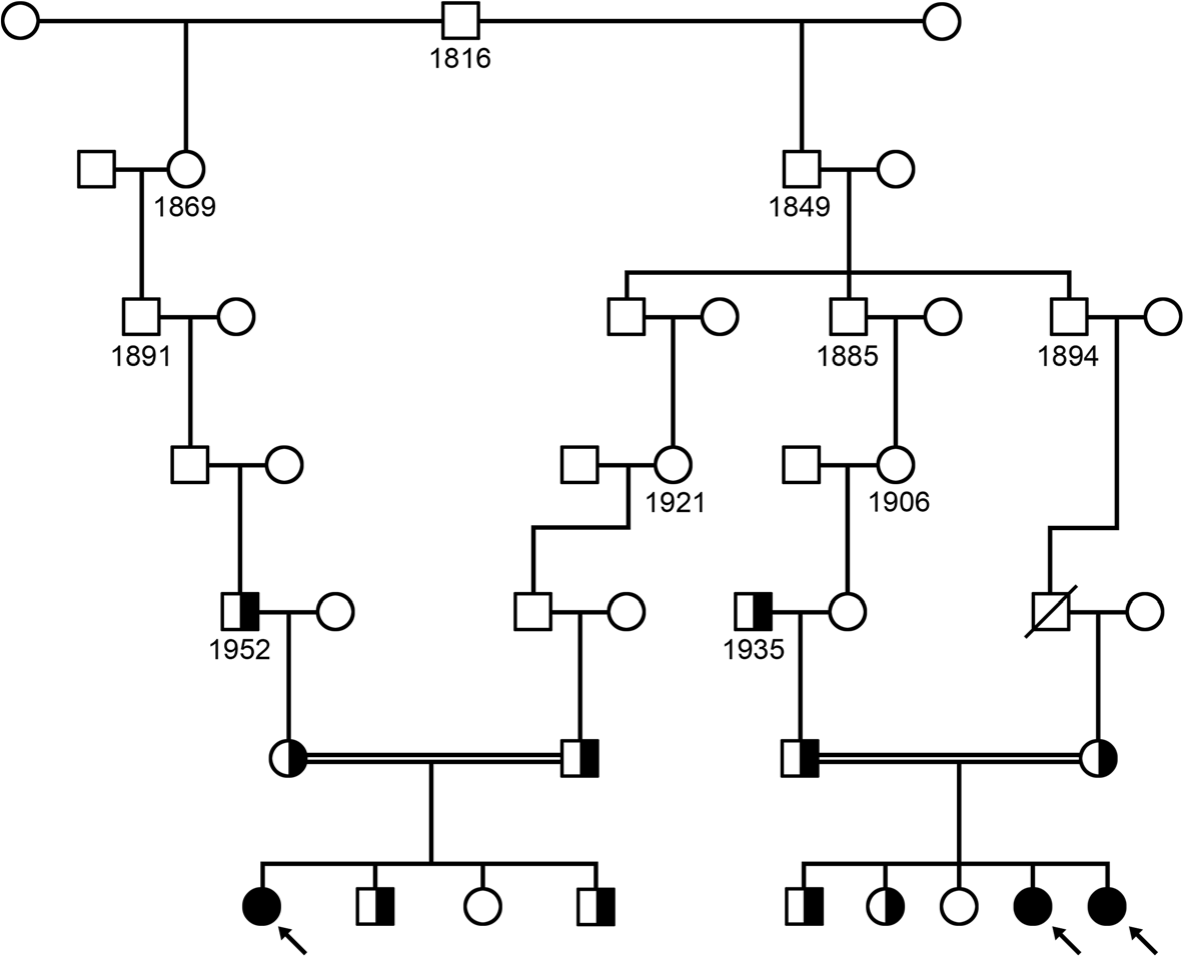
Ancestral pedigree depicting the relationship between three affected individuals. The common ancestor for three individuals homozygous (shaded symbols; designated with arrows) for the c.1624–11G>A mutation in *ZAP70* is an individual who was born in 1816. Individuals heterozygous for c.1624–11G>A are depicted as half-shaded circles and squares, and consanguineous marriages are depicted by double solid lines. Open circles in the bottom generation represent individuals who do not carry the *ZAP70* mutation. Not all individuals depicted were tested for the *ZAP70* mutation

**Fig. 2:**
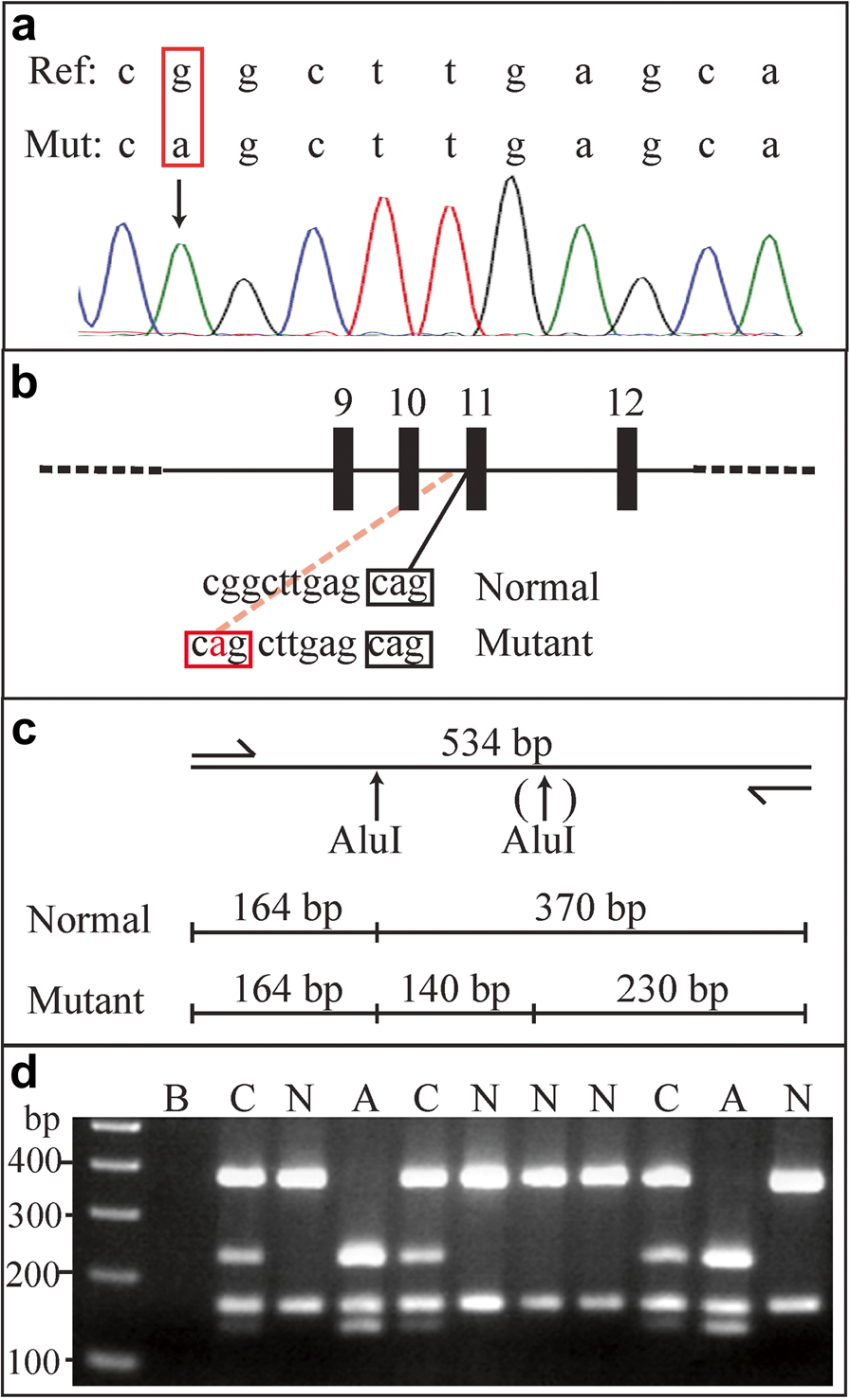
Analyzing the c.1624−11G >A mutation of *ZAP70*. Panel **a** - Sanger sequencing of genomic DNA from an affected individual depicting a homozygous intronic g >a mutation. Panel **b** – A schematic representation of a portion of the *ZAP70* gene indicating how the mutation generates a new acceptor sequence in affected individuals. Panel **c** – A restriction map of the PCR amplified product depicting the position of a second AluI cutting site (↑) in mutated alleles. Panel **d** - 534 bp PCR products were digested with AluI and the resulting fragments separated by agarose gel electrophoresis. Lane 1 is a 100 bp DNA standard and Lane 2 is a water blank (B). Lanes 3 through 12 depict fragments generated from individuals of normal (N) genotype (370 bp and 164 bp), affected (A) genotype (230 bp, 164 bp and 140 bp) or carrier (C) genotype (370 bp, 230 bp, 164 bp and 140 bp)

The recognition of an apparent increased frequency of immunodeficiency among Mennonite children in Manitoba [9] provided the impetus to search for the cause of this disorder in three affected individuals and their immediate families (pedigree depicted in Fig. 1). Each of the affected individuals depicted descend from a common ancestor, born in 1816. Initially, microsatellite markers surrounding genes known to cause primary immunodeficiency were analyzed in these families. All three affected individuals were identically homozygous for six markers flanking *ZAP70* (data not shown). Subsequently, Sanger sequencing (Fig. 2a) of DNA from these three affected individuals revealed that all were homozygous for the previously defined [2] *ZAP70* mutation (c.1624–11G>A). The extended families of the other four affected individuals from this study also display consanguinity loops (pedigrees not shown), but a single ancestor common to all seven affected individuals was not identified.

Because the causative mutation, c.1624–11G>A in *ZAP70* has been shown to cause protein deficiency underscoring the phenotype [2–5], we developed a PCR-based assay to determine the genotypes of related and unrelated Mennonite individuals. The g to a intronic mutation (Fig. 2b and c) creates an AluI site (ggct to agct) that allows carriers, non-carriers and affected individuals to be readily differentiated (Fig. 2d). Genotyping was conducted on genomic DNA isolated from whole or cord blood, or buccal swab. All genotypes determined from cord blood were confirmed with a peripheral blood sample, as a way to exclude possible maternal contamination of the cord sample. In all cases genotypes of the peripheral sample were concordant with those determined on the cord sample. Of the 125 study subjects, 79 typed as normal (GG), 39 as carriers (AG), and 7 as affected (AA). In addition, we determined *ZAP70* genotypes for 115 random individuals, all of whom typed as normal, and also from ten random individuals who self-identified as of Mennonite descent, one of whom genotyped as a carrier.

## Discussion

Because immune deficiencies in children should be considered pediatric emergencies, early diagnosis and treatment [8, 10] is the very best option for affected individuals. However, recognizing immune deficiency caused by a loss of function mutation in *ZAP70* can be difficult and even delayed because most patients have detectable lymphoid tissue, normal lymphocyte counts and immunoglobulin levels [9, 11, 12]. Additionally, the age of presentation can vary from about 4–17 months, and in the case of Mennonites, affected children living in rural communities may not be referred to the pediatric tertiary care center in a timely manner.

Our study underscores the importance of developing a targeted screening test for ZAP-70 kinase deficiency in Mennonites and for engaging the community at the onset. All of our study subjects belong to the same genetic isolate and can trace their roots to a group of immigrants arriving to Canada in the 1870s. Because of a high birth rate and large family size, the founding c.1624–11G>A allele of *ZAP70* has been maintained in the current population. Our identification of 39 carriers among 125 study subjects is not surprising since most participants were directly related to an affected individual, but the identification of a carrier in 10 random Mennonite samples is concerning. We do acknowledge that estimates of carrier allele prevalence using just 10 samples may not accurately reflect the true frequency, but finding random samples for such calculations in a closed population is impossible. Despite these limitations, we have identified affected individuals in multiple families, suggesting that the c.1624–11G>A allele of *ZAP70* is frequent enough in the Canadian Mennonite community. Further, because members of this cohort have moved to Mexico (including one of the families we studied with two affected children), Belize, Paraguay and other South American countries, screening for the c.1624–11G>A allele of *ZAP70* should also be undertaken in these countries.

## Conclusion

The development of a definitive screening test for ZAP-70 kinase deficiency in Mennonites will ensure prompt diagnosis and treatment for affected children, and will also provide pregnancy counselling opportunities for at risk couples in Canada and around the world.
